# Haplotype Variations and Evolutionary Analysis of the Granule-Bound Starch Synthase I Gene in the Korean World Rice Collection

**DOI:** 10.3389/fpls.2021.707237

**Published:** 2021-08-24

**Authors:** Thant Zin Maung, Ji-Min Yoo, Sang-Ho Chu, Kyu-Won Kim, Ill-Min Chung, Yong-Jin Park

**Affiliations:** ^1^Department of Plant Resources, College of Industrial Sciences, Kongju National University, Yesan-gun, South Korea; ^2^Center of Crop Breeding on Omics and Artificial Intelligence, Kongju National University, Yesan-gun, South Korea; ^3^Department of Applied Life Science, Konkuk University, Seoul, South Korea

**Keywords:** granule-bound starch synthase 1, haplotype, SNP, domestication, cultivated rice, wild rice

## Abstract

Granule-bound starch synthase I (*GBSSI*) is responsible for *Waxy* gene encoding the, which is involved in the amylose synthesis step of starch biosynthesis. We investigated the genotypic and haplotypic variations of *GBSSI* (*Os06g0133000*) gene, including its evolutionary relatedness in the nucleotide sequence level using single-nucleotide polymorphisms (SNPs), indels, and structural variations (SVs) from 475 Korean World Rice Collection (KRICE_CORE), which comprised 54 wild rice and 421 cultivated represented by 6 ecotypes (temperate japonica, indica, tropical japonica, aus, aromatic, and admixture) or in another way by 3 varietal types (landrace, weedy, and bred). The results revealed that 27 of 59 haplotypes indicated a total of 12 functional SNPs (fSNPs), identifying 9 novel fSNPs. According to the identified novel fSNPs, we classified the entire rice collection into three groups: cultivated, wild, and mixed (cultivated and wild) rice. Five novel fSNPs were localized in wild rice: four G/A fSNPs in exons 2, 9, and 12 and one T/C fSNP in exon 13. We also identified the three previously reported fSNPs, namely, a G/A fSNP (exon 4), an A/C fSNP (exon 6), and a C/T fSNP (exon 10), which were observed only in cultivated rice, whereas an A/G fSNP (exon 4) was observed exclusively in wild rice. All-against-all comparison of four varietal types or six ecotypes of cultivated rice with wild rice showed that the *GBSSI* diversity was higher only in wild rice (π = 0.0056). The diversity reduction in cultivated rice can be useful to encompass the origin of this gene *GBSSI* during its evolution. Significant deviations of positive (wild and indica under balancing selection) and negative (temperate and tropical japonica under purifying selection) Tajima's *D* values from a neutral model can be informative about the selective sweeps of *GBSSI* genome insights. Despite the estimation of the differences in population structure and principal component analysis (PCA) between wild and subdivided cultivated subgroups, an inbreeding effect was quantified by *F*_*ST*_ statistic, signifying the genetic relatedness of *GBSSI*. Our findings of a novel wild fSNPS can be applicable for future breeding of waxy rice varieties. Furthermore, the signatures of selective sweep can also be of informative into further deeper insights during domestication.

## Introduction

Starch, which serves as a reserve carbohydrate in plants, is a major food component for humans worldwide. It exists in the form of granules, which are composed almost entirely of two major polysaccharides: amylose and amylopectin (Bertoft, 2017). Eating and cooking qualities (ECQs) are the main parameters of rice that influence consumer preferences and are used to designate special-quality rice. The amylose content (AC) in rice controls its ECQs (Tian et al., 2009; Phing Lau et al., 2016) and is considered as a major attribute (Fitzgerald, 2010). High-amylose (after cooking) rice varieties have nutritional benefits because of their slow digestibility (Tao et al., 2019), whereas low-amylose rice contains a large proportion of rapidly digestible starch that results in a fast increase and simultaneously a quick decrease in blood glucose levels, which in turn contributes to obesity, diabetes, and cardiovascular diseases (Lehmann and Robin, 2007).

The AC in rice is determined by functional mutations, which alter the main characteristics of not only sticky varieties but also non-sticky varieties (Olsen et al., 2006). Based on waxy DNA sequencing, Olsen et al. (2006) reported the mutations that were present in temperate japonica rice but seemed to be rare or lacking in varieties such as tropical japonica, indica, aus, and aromatic. Amylose biosynthesis is regulated by an enzyme called granule-bound starch synthase I (*GBSSI*), which is encoded by the waxy gene (Shure et al., 1983; Sano et al., 1985; Chao et al., 1989; Wang et al., 1990; Yamamori et al., 1994), and the AC in rice after milling can be categorized as waxy (1–2%), very low (5–12%), low (12–20%), intermediate (20–25%), or high (>25%) (Bao, 2012). *GBSSI*, one of the *GBSS* isoforms expressed in seed embryos (Shure et al., 1983), is located on chromosome 6, whereas another isoform located on chromosome 7, *GBSSII*, functions in non-storage tissues such as leaves (Dian et al., 2003; Hirose and Terao, 2004). However, quantitative trait locus (QTL) mapping confirmed that the AC in rice is largely controlled by the *GBSSI* locus (He et al., 1999; Aluko et al., 2004; Fan et al., 2005) although there has been a recent report on different ECQ controlling genomic regions occupying several detected QTLs for amylose and protein contents other than *GBSSI* (Hori et al., 2021). High amylose originates from wild rice (*Oryza rufipogon*) and is also found in the tropical japonica and indica varieties, which are classified as South and Southeast Asian varieties (Morishima and Sano, 1992), whereas low amylose is generally preferred in Northeast Asia and is found in the temperate japonica variety (Juliano, 1992). These different origins of different amylose contents rice maybe because of different consumers' preference by regions, for example, low-amylose in Vietnam and some provinces of China, intermediate-amylose in Iran, Pakistan, Malaysia and the Philippines, and high-amylose in Myanmar and Sri Lanka, respectively (Calingacion et al., 2014). Like rice, in East Asia, mainly Korea and China, and Southeast Asian countries, glutinous maize production has been increasing due to a high consumption for its special waxy type (Sa et al., 2015) as well as the consumption pattern shifts from a traditional rice-based diet to a Western meat-based diet (Kim H. R. et al., 2021). Unlike the waxy locus of rice, maize has a higher genetic variation level within its waxy gene region (Huang et al., 2010). However, the genetic differentiation of a waxy gene region of both glutinous rice and maize was lower than those of non-glutinous ones (Olsen and Purugganan, 2002; Yamanaka et al., 2004; Fan et al., 2008, 2009; Wei et al., 2012; Zheng et al., 2013; Sa et al., 2015; Luo et al., 2020).

Different waxy alleles are widely distributed throughout the world (Zhang et al., 2019) despite the selection of people on different waxy alleles and their cultural preferences (Calingacion et al., 2014). Previous studies described two important waxy alleles, *Wx*^*a*^ (22–29% AC in indica rice) and *Wx*^*b*^ (12–19% AC in japonica rice) in rice (Sano et al., 1985; Wang et al., 1995). However, because these two alleles were not enough to explain much of the variation in AC (Mikami et al., 2008), many rice research groups have discovered and reported numerous other functional waxy alleles (Cai et al., 1998; Isshiki et al., 1998; Wanchana et al., 2003; Mikami et al., 2008; Yang et al., 2013) in connection with amylose, proving their close relatedness to *GBSSI*. Among them, *Wx*^*Iv*^ is an ancestral waxy allele from wild rice that was recently demonstrated by a Chinese research group to be involved in domestication by artificial selection (Zhang et al., 2019). Moreover, the origin of a new novel waxy allele *Wx*^*Ia*^ (a combined mutation of *Wx*^*in*^ and *Wx*^*b*^) provided new insights of a unique phenotype into rice quality improvement (Zhou et al., 2021), and the other five old functional waxy alleles, namely, *Wx*^*b*^*, Wx*^*mp*^*, Wx*^*op*^*, Wx*^*in*^, and *Wx*^*a*^, identified in their sequencing analyses are distinguishable by their specific polymorphic sites in intron 1 and exons 2, 4, 6, and 10, respectively (Zhang et al., 2019). As an additional consequence, a new rare transgenic waxy allele “*Wx*^*mw*^” exhibited by a moderate *GBSSI* was also introduced, resulting in a moderate AC (Zhang et al., 2021). Therefore, it can be assumed that several functional waxy alleles have been characterized for their different major polymorphic sequences (Zhang et al., 2021). For maize waxy alleles, many mutant alleles have been identified (more than 50 alleles) (Huang et al., 2010), and most were insertions or deletions (Huang et al., 2010; Xiaoyang et al., 2017), representing the most recent mutant allele by *wx-Reina* in Chinese waxy maize (Wu et al., 2019).

Much research has been performed on rice evolution over its long history (Khush, 1997). Various strategies, such as exploring wild rice germplasm and natural or artificial mutations, have also been employed by breeders to broaden the genetic diversity of rice (Shen et al., 2017). Recent research was performed on the breeding improvement of some ECQ Korean rice by marker-assisted backcrossing (MABc) markers [kompetitive allele-specific PCR markers (KASP)] (Kim M.-S. et al., 2021), the development of novel waxy alleles by CRISPR/Cas9 system for the improvement of fine-tuned AC rice (Huang et al., 2020), and CRISPR/Cas9-based editing of the waxy gene in a 5′ untranslated region- (UTR-) intron region for rice grain quality improvement (Zeng et al., 2020). Different selection methods for detecting genetic variations (alleles) were also increasing for their patterns or distinctive signatures and then determined signatures whether due to selection or other confounding effects such as bottlenecks, expansions, and subdivided populations (Sabeti et al., 2006). Because the preferences of people for waxy rice have been implied in many different ways (Calingacion et al., 2014), the genetics and functional roles of waxy alleles are still highly variable (Yu et al., 2011; Huang et al., 2012; Zhao et al., 2018). As a consequence, the origins of different waxy alleles during evolution and domestication were highly varied depending upon their corresponded mutations and cultivation of selected varieties. Taking this into account, we conducted haplotype variations and evolutionary analysis of *GBSSI* in Korean World Rice Collection (KRICE_CORE) to detect the functional alleles *GBSSI*, its genetic diversity, population differentiation, and evolutionary relationship among the classified rice groups. Our results will contribute to rice breeding scientists, an understanding of the domestication signatures of waxy alleles related to their haplotypes as well as their distribution patterns among the classified populations, which in turn promotes the breeding program of new waxy rice cultivars.

## Materials and Methods

Many functional studies of *GBSSI* employing genetic linkage mapping have been carried out annually around the world. Nevertheless, the different alleles of *GBSSI* and their functional responses are different due to their domesticated ancestors in the group of rice accessions. In this study, we used 475 Korean rice accessions (KRICE_CORE) for the genetic identification of *GBSSI* in terms of diversity and relatedness among and between the classified subgroups as well as performing additional validation of functional waxy alleles. A series of analyses were conducted as follows.

### Plant Materials and Experimental Site

A heuristic set of 421 rice accessions, which were represented by 3 original variety types (landraces, weedy varieties, and bred varieties) (Supplementary Data 1), previously collected from global varieties, and generated by the National GenBank of the Rural Development Administration (RDA-GenBank, Republic of Korea) using the PowerCore program (Kim et al., 2007), was selected for whole-genome resequencing (Kim et al., 2016). Additional set of 54 wild rice accessions were also shared by the International Rice Research Institute (IRRI) in 2017.

For these 421 Asian cultivated and 54 wild rice accessions, field experiments were conducted in the departmental field of the Plant Resources Department, Kongju National University (Yesan Campus), in 2016 and 2017. Within these three originally cultivated varietal types, there were 6 ecotypes, including 279 temperate japonica, 26 tropical japonica, 102 indica, 9 aus, 2 aromatic, and 3 admixed varieties (Supplementary Table 1). Recommended cultural practices for crop management were carried out as necessary.

### DNA Extraction, Resequencing, and Variant Calling

Plant samples (young green leaves) were collected ~15 days after the transplantation from all tested plants. DNA extraction was performed by the cetyltrimethylammonium bromide (CTAB) method, and the genomic DNA was stored in a refrigerator at 4°C until use (Doyle and Doyle, 1990). Qualified DNA was used for whole-genome resequencing of the collected rice varieties with an average coverage of ~15X on the Illumina HiSeq 2000 Sequencing Systems Platform, and these HiSeq 2000 sequencing data were deposited in the NCBI GenBank database (accession number, BankIt2477403: MZ514142–MZ514616). The decoded sequences were saved in the FastQ file format. VCFtools (variant call format) version 0.1.15 (Danecek et al., 2011) was used to remove missing values and heterozygotes in the raw data saved in the FastQ format. To compare the output sequences among the accessions, high-quality reads that remained after removing missing values and heterozygotes were aligned to the International Rice Genome Sequencing Project 1.0 (IRGSP) rice genome sequence (Kawahara et al., 2013). The alignment of the reads was saved in a binary alignment map (BAM) format. Duplicate reads aligned in multiple locations were removed using PICARD (version 1.88) (Toolkit, 2019). Then, the single-nucleotide polymorphism (SNP) and insertion/deletion (InDel) calling was performed using the Genome Analysis Toolkit (GATK) (version 4.0.1.2) (DePristo et al., 2011; Van der Auwera and O'Connor, 2020) to find the SNP regions in the BAM file. The extracted mutations were saved in a VCF file format, and filtering was commanded in VCFtools (version 0.1.15) to remove false-positive SNPs/InDels, using the most common criteria: a minor allele frequency (MAF) of 0.02 and a maximum missing data ratio (MDR) of 0.3. To identify the genetic variants, we viewed the specific variant files of the classified subgroups by using bcftools version 1.8 and counted their respective number of genetic variants in the TASSEL 5 version 20210408 (Bradbury et al., 2007).

### Population Structure, Principal Component Analysis, and Fixation Index (*F_*ST*_* Test)

To identify the existence of number of populations in 475 rice accessions, VCFtools version 0.1.15 was first used to convert the previously called variants into a plink output, and using the PLINK version 1.07 analysis toolset, bed files were created again. Two additional files (.bim and.fam format) were incorporated by using a Python script (structure.py) with fastStructure (Raj et al., 2014) package tools and a range of increasing *K*-values from 2 to 7. The admixture patterns of defined populations (population structure) were inferred using average *Q*-values by the POPHELPER version 2.3.1 (Francis, 2017) analytical tool in RStudio version 1.4.1106. To multivariate the original data sets of *GBSSI* genetic variants by their similarities or differences among the identified subpopulations, we conducted principal component analysis (PCA) displaying as a dimensional scale in RStudio version 1.4.1106. The list of principal components (PC1 and PC2) referring to variants was generated in TASSEL 5 version 20210408 (Bradbury et al., 2007), and the relatedness among the classified subpopulations was investigated in two-dimensional (2D) scatterplots, RStudio version 1.4.1106.

### Nucleotide Diversity and Tajima's *D*

To determine the degree of polymorphism within the pairwise comparison of classified populations, we investigated their diversity by nucleotide diversity values (π). We calculated Tajima's *D* values to measure the difference between the estimated average number of nucleotide differences and the observed number of segregating sites for all 475 rice accessions. To observe the performance of genetic relationship between and among the populations, we further investigated a fixation index (*F*_*ST*_ values). Using VCFtools version 0.1.15, variant files were selected for the *GBSSI* gene region for the classified representative cultivated ecotypes/subpopulations to be compared. The sliding window sizes used for nucleotide diversity (π) and Tajima's *D* calculations were the same, at 1.5 kb, and the values were compared among the classified rice groups based on their varietal types or ecotypes.

### Haplotype Network

A haplotype network was constructed to investigate the genetic relatedness of the analyzed samples based on their variants within the *GBSSI* gene region. VCFtools were used to specify the selected gene regions of the tested samples, and an alignment with the reference gene sequences adapted from RAP-DB (https://rapdb.dna.affrc.go.jp/index.html) was performed in the Molecular Evolutionary Genetics Analysis (MEGAX version 10.1.8) (Kumar et al., 2018). The compiled list of aligned sequences was saved in a nexus file format and then analyzed in DnaSP (version 6.0) (Rozas et al., 2017) to generate a list of haplotypes together with their accession numbers. Taking the accessions with each haplotype into account, the list of the same mutated sequences was created for each subpopulation/ecotype in Population Analysis with Articulate Trees (PopART) (Leigh and Bryant, 2015), and a TCS network (Clement et al., 2000) was drawn by using this ecotype list.

## Results

Firstly, variant types were categorized based on the observed number. Based on the results of an haplotype variation analysis, the functional positions of SNPs and InDels were identified and subjected to a diversity analysis to determine whether they had been under selection during domestication in cultivated or wild rice. The domestication was investigated through the signature of selective sweep in terms of calculating nucleotide diversity (π), Tajima's *D*, and *F*_*ST*_, which were analyzed together with statistical approaches such as population structure analysis and PCA. Using a haplotype list generated by different genetic variation analyses, networks were constructed to determine the genetic association of *GBSSI* between and among the classified ecotypes, including wild rice. Within the *GBSSI* gene region, the analyzed results revealed many differences between cultivated and wild rice that were accumulated during domestication.

### Identification of Genetic Variations

The genomic region of *GBSSI* was 1,765,622.,…,1,770,653 on chromosome 6. Within this *GBSSI* gene region, we performed variant calling to identify genetic variants and classified the results as SNPs, InDels, structural variations (SVs), and different variations (DVs) (Table 1). We counted the numbers of different variants based on the subpopulations to which they belonged. Among the analyzed results, we observed many more SNPs than any other variant type, and all subpopulations contained more SNPs than other variants. In addition, we separated the wild rice group, including *Oryza nivara* and *O. rufipogon* (which are the most commonly used wild ancestors), from the remaining groups. We generated a complete list of the genetic variant types according to the subpopulations to which they belonged, including this group of wild rice. The total variant number was higher in the wild group than in the cultivated group. Within the cultivated subpopulation, indica rice harbored the most SNP variants (35), followed by admixture (27) and aromatic (21) rice.

**Table 1:** Summary of genetic variations in the granule-bound starch synthase I (*GBSSI*) gene region of 475 accessions from the Korean rice collection.

**Group**	**Subgroup/Ecotype**	**No. of variations**				**No. accessions**
		**SNP**	**InDel**	**SV** ^**b**^	**DV** ^**c**^	
Cultivated rice	Temperate Japonica	6	0	1	2	279
	Tropical Japonica	16	2	1	9	26
	Indica	35	6	1	10	102
	Aus	2	5	0	8	9
	Aromatic	21	0	0	0	2
	Admixed	27	3	0	7	3
Wild rice	*O. nivara*	91	38	0	21	3
	*O. rufipogon*	20	5	0	6	3
	Others^a^	158	79	3	31	48

*The GBSSI reference gene region was adapted from Nipponbare*.

*Others^a^: a group of wild rice accessions other than Oryza nivara and Oryza rufipogon*,

*SV^b^: structural variation*,

*DV^c^: different variation*.

### Analysis on Haplotype Variations and Sequence Comparison

**Figure 1:**
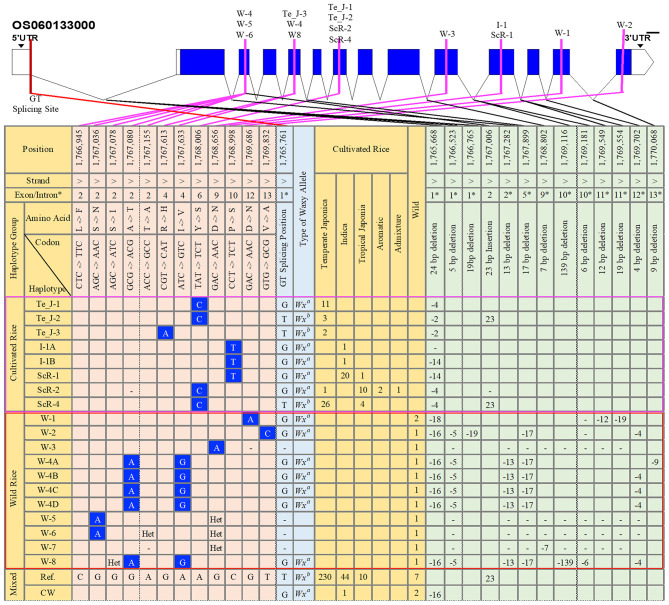
Gene structure and haplotype analysis of the granule-bound starch synthase I (*GBSSI*) (*Os06g0133000*) gene in 475 accessions of the Korean rice collection. The haplotypes were grouped into cultivated rice, wild rice, and mixed (cultivated rice and wild rice). Orange-colored columns provide the list of haplotypes together with their respective numbers of rice accessions in each subpopulation. Light-blue-colored columns indicate the corresponded waxy alleles based on the possession of a “G” or “T” nucleotide at the G-T splicing site of intron 1. Light-orange-colored columns indicate all single nucleotide polymorphism (SNP) variations found only at non-synonymous mutation sites. Light-green-colored columns represent variation sites for InDels. Blank cell indicates major alleles in that position. “Het” refers to “heterozygote,” and a dash (-) indicates the position for an “unknown” nucleotide or generally refers to “N” (any nucleotide A, T, G, or C). CW, cultivated and wild; Te_J, temperate japonica; I, indica; ScR, subcultivated rice; W, wild rice; Ref., reference. The selected number of chromosome positions and sorted haplotypes were based on the findings for functional SNPs (fSNPs).

To examine the haplotypic variations in terms of SNPs and InDels in the gene region of *GBSSI* (*Os06g0133000*), we conducted a haplotype analysis (Supplementary Data 2), investigating all identified functional alleles of *GBSSI* among the 475 tested rice accessions. The 475 genetically different sequences were aligned to the Nipponbare reference and validated for different functional variants. We found a total of 59 haplotypes for 43 SNPs and 70 InDel mutation sites (Supplementary Data 3). All these haplotypes were grouped according to the ecotypes that contained them, and we discussed only the functional haplotypes showing non-synonymous substitutions and InDels (Figure 1). In addition to functional haplotypes, we also included the reference haplotype (Ref.), found in the highest number of rice accessions, namely, 230 temperate japonica, 44 indica, 10 tropical japonica, and 7 wild rice accessions, and another combined haplotype of cultivated and wild rice (CW), in the figure.

Across the *GBSSI* gene region (position 1,765,622–1,770,653), we verified a total of 12 functional SNPs (fSNPs) located in exons 2, 4, 6, 9, 10, 12, and 13 among 475 diverse rice accession. For further insights, we specified three main haplotype groups, cultivated rice, wild rice (W), and their combination (cultivated and wild rice). According to the major findings for fSNPs, the haplotypes in the cultivated rice group were replaced by a parental ecotype or subcultivated haplotypes or haplotypes from temperate japonica (Te_J), indica (I), and subcultivated rice (ScR) (Figure 1). We identified three fSNPs only in the cultivated group: G/A (exon 4), A/C (exon 6), and C/T (exon 10). An fSNP substitution (A/C) in exon 6 belonged to only temperate japonica (Te_J-1 and Te_J-2), resulting in a tyrosine (Y) amino acid transition into serine (S), whereas Te_J-3 was a polymorphism (G/A) for an amino acid change of histidine (H) for arginine (R) in exon 4. Two indica haplotypes (I_1A and I_1B) exhibited a C/T replacement in exon 10 of the *GBSSI* gene region, resulting in an amino acid change from proline (P) to serine (S) by a C/T substitution. An additional fSNP (C/T) was also observed in the haplotype ScR-1, representing 20 indica, and 1 tropical japonica accessions. We found two more haplotypes, ScR-2 and ScR-4, showing the same polymorphism (A/C SNP) as that of temperate japonica haplotypes. In brief, we observed that the cultivated group had only three fSNPs (G/A, A/C, and C/T) in exons 4, 6, and 10, corresponding to different subpopulations of 73 rice accessions, namely 43 temperate japonica, 22 indica, 15 tropical japonica, 2 aromatic, and 1 admixture.

In the wild group, we observed six different positions showing fSNPs (Figure 1), which were all different from those identified in the cultivated group. There were 10 summarized wild haplotypes accounting for 4 G/A, A/G, and T/C fSNPs in exons 2, 9, 12, and 13. Of these haplotypes, W-1, W-2, and W-3 each had fSNPs, but we found that W-1 and W-3 were the same substitution of G/A for the amino acid change from aspartic acid (D) to asparagine (N), whereas the T/C substitution belonged to W-2. We observed two different fSNPs, G/A in exon 2 and A/G in exon 4 of these haplotypes, W-4A to W-4D and W-8. One more G/A SNP was detected in the two wild haplogroups (W-5 and W-6) at position 1,767,036, leading to an amino acid change from serine (S) to isoleucine (I). Interestingly, none of the common fSNPs were found for the cultivated and wild groups.

Considering the haplogroups classified by fSNPs, the number of positions with InDel variants was also selected for additional analysis of haplotype variations. The variation in differences categorized as InDels between the cultivated and wild groups were similar to those categorized as SNPs. For example, one InDel mutation (23 bp insertion) in exon 2 was identified only in the cultivated group, namely, in japonica (Te_J_1 and ScR-4). Similarly, different InDels at different positions in introns 1, 2, 5, and 12 were also observed only in the wild group. Specifically, W-1 showed three deletions (18, 12, and 19 bp) in introns 1 and 11. W-3, W-4 (A to D), and W-8 showed 16 and 5 bp deletions in intron 1, 13 bp deletions in intron 2, 17 bp deletions in intron 5, and 4 bp deletions in intron 12, respectively. One last finding from a haplotype analysis was a functional G to T mutation at the 5′ splice site of intron 1, which was a key to distinguishing waxy cultivars from nonwaxy cultivars.

### Population Structure, PCA, and Fixation Index (*F_*ST*_*Test)

**Figure 2:**
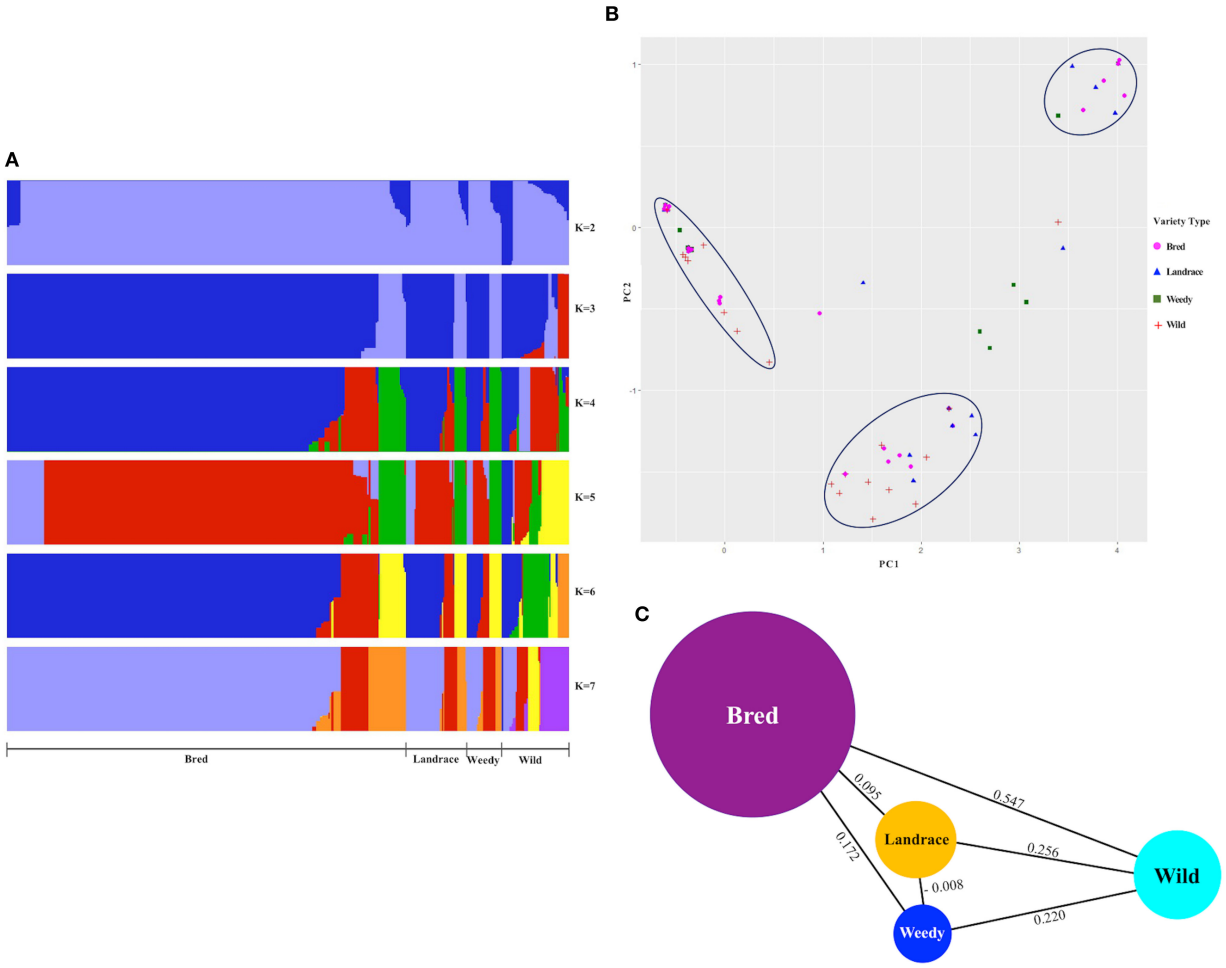
Estimation of structure and population differentiation within the gene region of *GBSSI* (*Os06g0133000*) in the Korean rice collection in terms of different varietal types (landrace, weedy, bred, and wild). **(A)** Population structure clustering of the *GBSSI* gene in 475 accessions of the Korean rice collection analyzed by increasing *K*-values from 2 to 7. For each *K*-value, different colors refer to different numbers of clustered populations. **(B)** Two-dimensional (2D) principal component analysis (PCA) of 475 accessions of the Korean rice collection. **(C)** Pairwise estimates of genetic differentiation (*F*_*ST*_ values) of the *GBSSI* gene among different variety types of the 475 accessions of the Korean rice collection.

We did a population structure to observe the differences by genetic variants among and between the classified cultivated subpopulations, including wild rice, and hence their genetic relatedness were verified (Figure 2A, Supplementary Figure 1). Admixed structure of cultivated varieties, landraces, weedy, and bred but a clear separation from wild varieties was observed at *K* = 2, and the remaining *K*-values, especially *K* = 6 and *K* = 7, created separate patterns of the classified types, but their internal clusters were mixed (Figure 2A). For *K* = 3 and *K* = 5, the structures of the wild varieties were differentiated from some of the cultivated varieties. According to the cluster patterns, the landrace, weedy, and bred groups showed similar structures with the close association of similar variants genetically identified within the *GBSSI* region. Once the classified populations were stratified by means of ecotypes (Supplementary Figure 1), we observed the mixed structure of indica with temperate japonica accessions at *K* = 3 and then with the wild group. At *K* = 4 and *K* = 7, the wild group was relatively differentiated from the temperate japonica and subsequently indica group shows its different structural pattern, and it was closely associated with the aus and admixed groups at *K* = 5. For most *K*-values (except *K* = 4 and *K* = 7), the population structure of cultivated ecotypes was more or less mixed with that of the wild groups, especially tropical japonica and indica. In other words, the wild group seemed to be closely related to many cultivated subpopulations in complicated patterns because of that most of internal subgroups of cultivated subpopulation were associated with each other and then their admixed populations could be parts of the wild during this gene, *GBSSI* domestication.

The PCA for the descriptive components of the landrace, weedy, wild, and bred varieties (Figure 2B) revealed that all the classified variety types were admixed in some proportion. Despite clear separations as groups, the classified varieties were associated especially among the cultivated groups. Wild varieties were clearly separated as a group but closely associated with most bred. The most diverse variety groups were landrace and weedy, whereas the bred varieties were closely associated with all other classified groups (landrace, weedy, and wild). When the values were multivariate in terms of classified ecotypes (Supplementary Figure 2) and descriptive together with wild, similar diverse patterns to varieties appeared, indicating that the values were distantly associated within each ecotype. Although indica ecotype indicated its clear separation by some proportion, its additional accessions and all the analyzed ecotypes were mixed together as well as with the wild rice accessions. In brief, the cultivated ecotypes, temperate japonica, and tropical japonica were closely associated with the wild.

To verify the level of genetic differentiation of *GBSSI* between the classified subgroups, we further calculated *F*_*ST*_ values. The greater the genetic distance between the populations, the less the breeding efficiency between them, and the more isolated they will be from another. We calculated the *F*_*ST*_ values of pairwise comparisons of the variety types, namely, landraces, weedy rice, bred rice, and wild rice (Figure 2C). We observed the highest average *F*_*ST*_value (0.547) between the wild and bred rice, followed by that (0.256) for the landrace-wild comparison and then that between landraces and weedy rice (−0.008). All cultivated varieties indicated their lower *F*_*ST*_ values among and between themselves than their values from the wild. We estimated *F*_*ST*_ values also for pairwise comparisons of cultivated ecotypes together with wild rice (Table 2). The values in this analysis were in a range from −0.172 between tropical japonica and aromatic rice to 0.747 between temperate japonica and aus rice. The *F*_*ST*_ value (0.729) between aromatic and aus rice was higher than the other *F*_*ST*_values. Despite the highest *F*_*ST*_ value (0.547) between the wild and landrace variety types, some cultivated ecotypes showed sizeable pairwise distances from wild rice, such as 0.703 (temperate japonica), 0.524 (aromatic), and 0.510 (tropical japonica). Interestingly, the classified cultivated subgroups were admixed with the wild rice in PCA, but in some classified subgroups, especially some ecotypes, their genetic distances were considerably distant from the wild in *F*_*ST*_ test.

**Table 2:** Pairwise estimates of genetic differentiation (*F*_*ST*_ values) of the *GBSSI* gene between different subgroups of 475 Korean rice collection accessions.

**Subgroups**	**Te_J**	**Tr_J**	**Indica**	**Aus**	**Aromatic**	**Admixture**	**Wild**
Te_J^a^	–						
Tr_J^b^	0.229	–					
Indica	0.470	0.274	–				
Aus	0.747	0.588	0.181	–			
Aromatic	0.459	−0.172	0.129	0.729	–		
Admixture	0.617	0.277	−0.136	0.400	0.201	–	
Wild	0.703	0.510	0.271	0.324	0.524	0.260	–

*Te_J^a^, temperate japonica*;

*Tr_J^b^, tropical japonica*.

### Nucleotide Diversity Analysis

**Figure 3:**
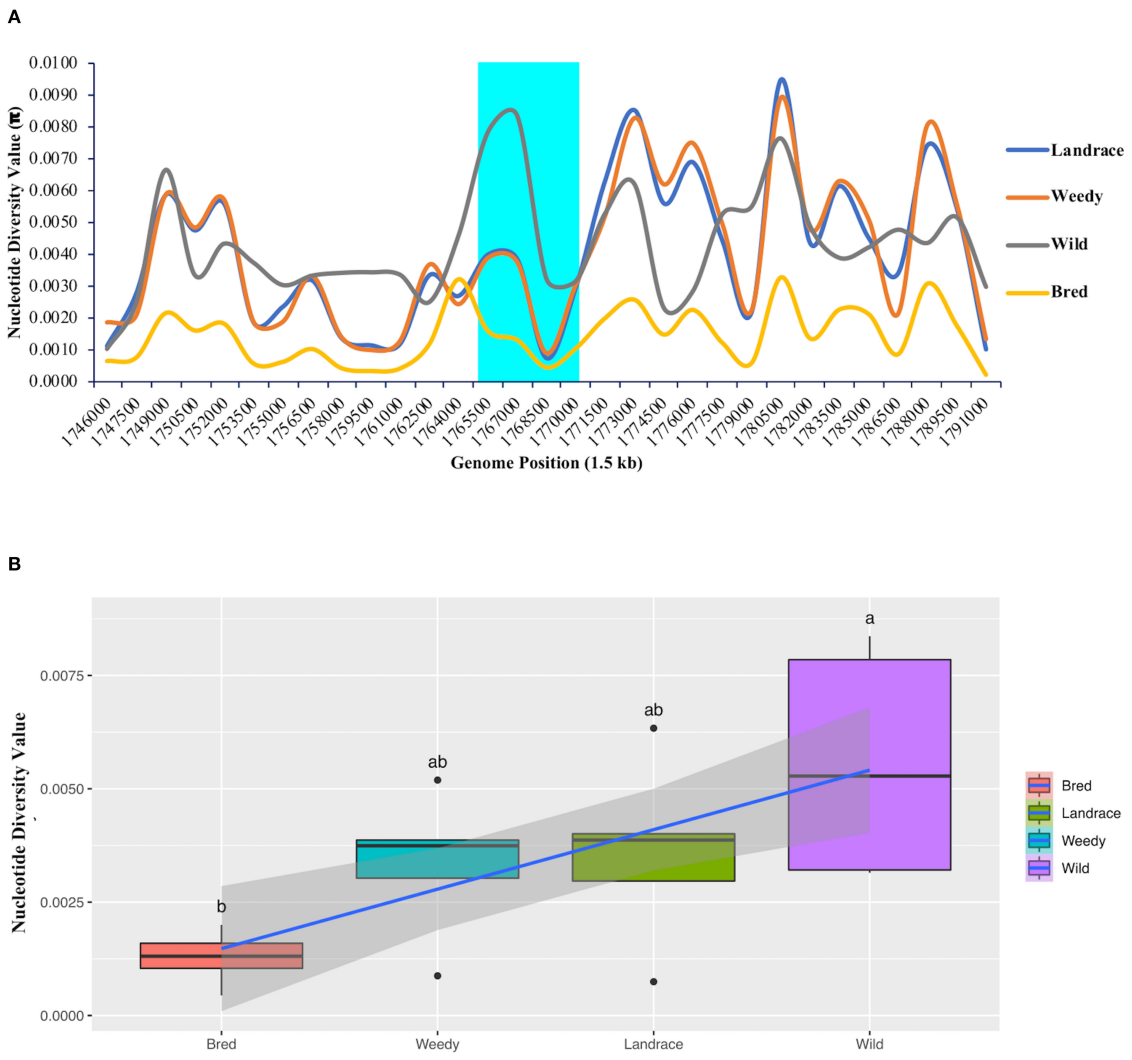
Nucleotide diversity analysis of *GBSSI* (*Os06g0133000*) in 475 accessions of the Korean rice collection by means of variety types (landrace, weedy, bred, and wild). **(A)** Nucleotide diversity (π-value) representing the number of nucleotide variations at individual segregating sites in 1.5 kb sliding windows within the *GBSSI* gene region. Cyan indicates the *GBSSI* gene region, and each colored line represents a different rice variety type. **(B)** Box plots representing the different distribution patterns of *GBSSI* genetic variations based on the mean nucleotide diversity values among the variety types.

An analysis of nucleotide diversity was performed to measure the degree of polymorphism among the classified groups or subpopulations that occurred at different segregating sites in the *GBSSI* gene region. In a comparison of the wild and cultivated subgroups (landraces, weedy varieties, and bred varieties), we found the highest nucleotide diversity value (π-value) in the wild group. All the calculated diversity values (π) at the fixed segregation sites in *GBSSI* region were < 0.0100 (Figure 3A). Within the *GBSSI* gene region, the identified represented position with the highest diversity value was 1,767,000, with the highest value in the wild subgroup (0.0084), followed by landraces (0.0039), weedy varieties (0.0037), and bred varieties (0.0013). A comparison of the average diversity values of these groups (Figure 3B) led to the same finding: the groups ranked in an ascending order as wild varieties, landraces, weedy varieties, and bred varieties, with the diversity values of 0.0056, 0.0036, 0.0033, and 0.0013 (Supplementary Table 2), respectively, indicating different ranges of nucleotide diversity values. Wild varieties showed a very wide range of nucleotide diversity values with a normal symmetric distribution while the distributions for the landraces and weedy varieties were negatively skewed because of a low variation at the lower end of the distribution.

To further evaluate the differences in the nucleotide diversity values among the ecotypes (Supplementary Figure 3A), we calculated their diversity values. According to the results, the highest degree of polymorphism was found at the represented position of 1,767,000 by 1.5 kb sliding window with admixture ecotype showing the highest value (0.0067) after the wild subgroup and temperate japonica showing the lowest value (0.0004). Similarly, among the classified cultivated subgroups, the average diversity values ranged from 0.0003 in temperate japonica rice to 0.0049 in admixed rice. The calculated values for the remaining three subgroups, namely, indica, aus, and temperate japonica, were 0.0044, 0.0016, and 0.0010, respectively (Supplementary Table 3) and in brief, all the classified ecotypes showed lower in diversity by a narrow range of diversity values within each ecotype (Supplementary Figure 3B). According to our calculated values, the wild group had the higher nucleotide diversity than all of the cultivated subpopulations (ecotypes). Among the cultivated ecotypes, japonica, especially temperate japonica, had low diversity at every comparison, suggesting that people selected these japonica varieties because of their lower polymorphism during the domestication of *GBSSI*.

### Tajima's *D* Test

**Figure 4:**
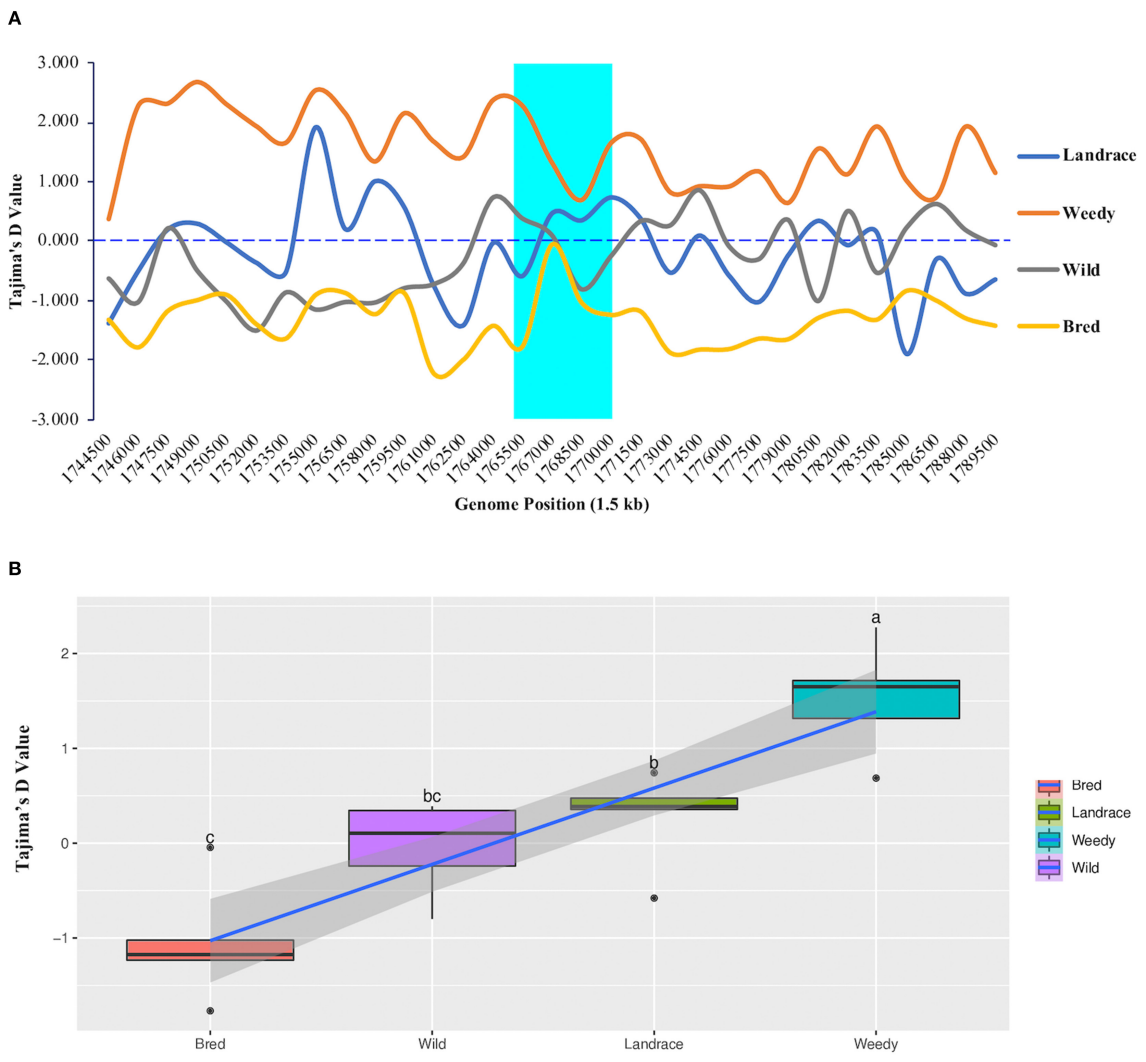
Tajima's *D* values of *GBSSI* (*Os06g0133000*) in 475 accessions of the Korean rice collection by means of variety types (landrace, weedy, bred, and wild). **(A)** Tajima's *D* values representing different individual segregating sites in 1.5 sliding windows of the *GBSSI* gene region. Cyan indicates the *GBSSI* gene region, and each colored line represents a different rice variety type. **(B)** Box plots represent different distribution patterns of *GBSSI* genetic variations according to Tajima's *D* values among the variety types.

To measure the difference between the estimated average number of nucleotide differences and the observed number of segregating sites, we calculated Tajima's *D* values for all 475 rice accessions. According to the resulting values within the *GBSSI* gene region, the weedy group had the highest Tajima's *D* value (2.276) at 1,765,500 (Figure 4A), and it was the only group with positive Tajima's *D* values at every analyzed position. In contrast, in the bred group, all the positions showed negative Tajima's *D* values, whereas landrace and wild groups showed both negative and positive values. In the comparison of their average values, the weedy group showed the highest positive D value (1.5286), followed by the landrace group (0.2764), and the lowest value was observed in the bred group (−1.0488) (Supplementary Table 2). The weedy and wild groups showed a relatively wide range of values compared to those of the landrace and bred groups, which were skewed positively (Figure 4B).

Once the values were calculated for ecotypes (Supplementary Figure 4A), we found that indica accessions had the highest Tajima's *D* value (2.6890) at the genome position of 1,768,500, and the lowest value was observed in tropical japonica accessions (−2.0771) at the genome position of 1,770,000. Within *GBSSI* genomic region, indica and admixture indicated only positive Tajima's *D* values whereas the remaining ecotypes showed both positive and negative Tajima's *D* values based on the identified positions. We compared the classified ecotypes according to their average D values, which revealed that indica accessions had the highest D value (1.8349) (Supplementary Table 3) and tropical japonica had the lowest value (−1.0801) while the rest of the ecotypes were in an order of admixed (0.9264), aus (0.4665), wild (−0.0403), and temperate japonica (−0.3401), with indica and temperate japonica accessions showing negatively skewed distributions of D values (Supplementary Figure 4B). In the comparisons of variety types and ecotypes, the wild group was followed by the japonica subpopulation in terms of lower Tajima's *D* values.

### Haplotype Network

**Figure 5:**
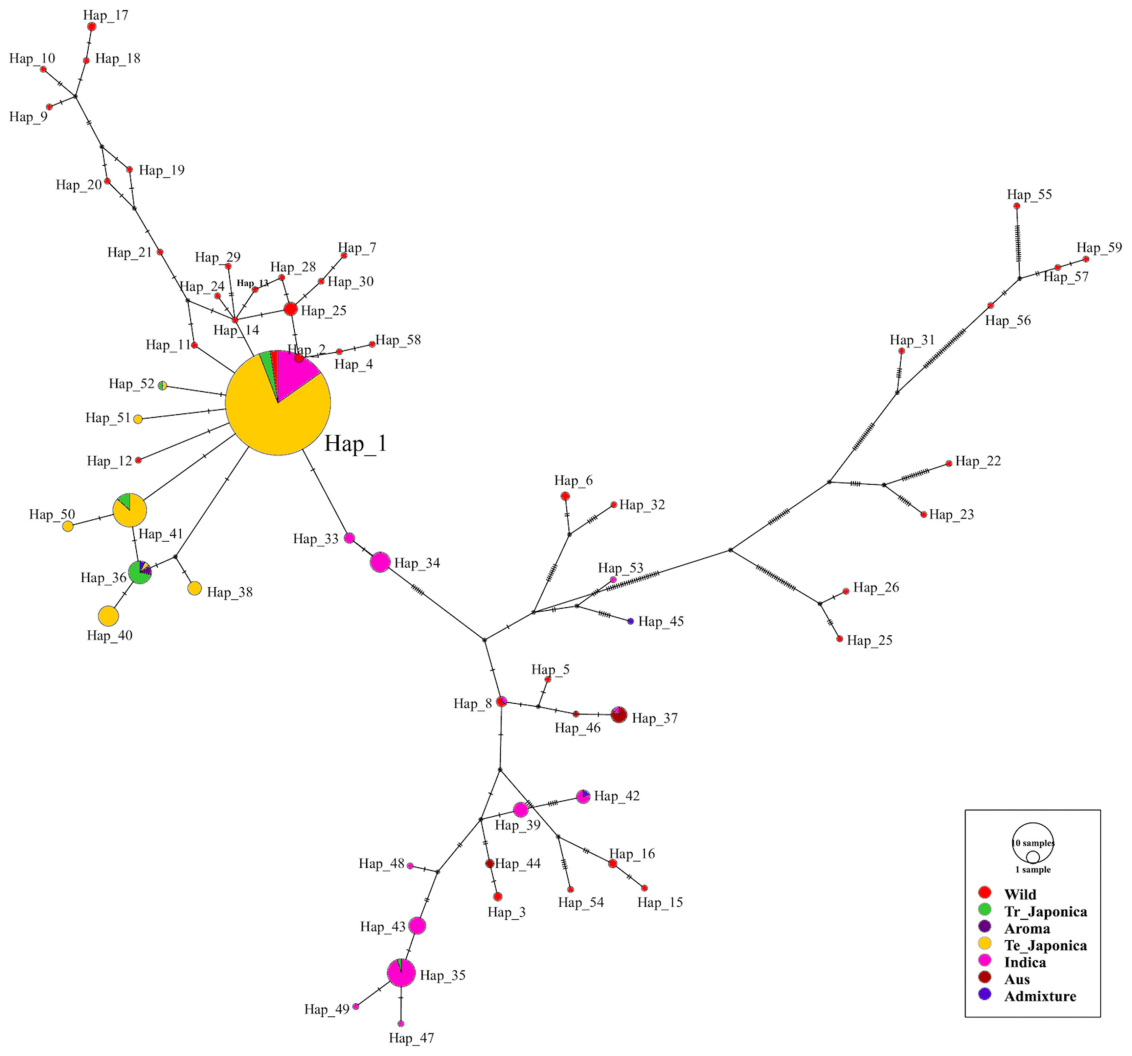
Haplotype network visualizing the evolutionary relationships between different genotypes of the *GBSSI* gene in 475 accessions of Korean rice collection. The size of each circle is proportional to the number of accessions that harbor the haplotype, and different colors refer to the ecotypes. The median vector indicated by the black circular dot is a hypothetical sequence used to connect the existing similar sequence (Bandelt et al., 1999). In the present analysis, there were 20 median vectors in total.

To visualize the genetic relatedness of the classified subpopulations of all tested rice accessions, we constructed and analyzed a haplotype network (Figure 5). To cover all the functional variants of *GBSSI*, we used the entire *GBSSI* region for a haplotype network construction. After a series of analyses, we identified 59 haplotypes (hereafter referred to as “Hap”) belonging to the classified ecotypes. According to the network, Hap_1 was a major haplotype (291 accessions), indicating the close genetic relatedness of *GBSSI* in most temperate japonica (230), indica (44), tropical japonica (10), and wild (7) rice accessions. As shown in figure, most of the haplotypes belonging to cultivated subpopulations are closely associated, with a few mutational steps (haplotypes 2, 11, 12, 14, 41, 51, and 52 are the derivatives of the major haplotype Hap_1). Moreover, there were additional predominant haplotypes composed of identical genomic sequences, e.g., Hap_41, belonging to temperate and tropical japonica accessions and Hap_36, belonging to tropical japonica, temperate japonica, aromatic, and admixed accessions. However, some haplotypes belonging to cultivated indica, admixed, and aus accessions were very far in terms of *GBSSI* genetic distance from japonica haplotypes. Most of the wild haplotypes were diverse in the *GBSSI* network and not only distant from the cultivated haplotypes but also separated from each other. This diverse display of wild rice indirectly indicated that wild rice accessions had different rare alleles of *GBSSI*.

## Discussion

In recent years, high-throughput sequencing technologies have been effectively employed in the resequencing of different sets of rice accessions for their genomics, evolutionary analyses, and functional genomics studies (Guo et al., 2014) to explore the diversity of rice genes in terms of their effects on agronomic traits (3,000 Rice Genomes Project, 2014; Huang et al., 2015; Yano et al., 2016). Evidence of strong selection was also important for determining the diversity level of *GBSSI* among cultivated subpopulations resulting from domestication (Yu et al., 2011). An association between functional markers (SNPs or InDels) and *Wx* gene expression (amylose biosynthesis) in rice has been described by many research groups, where the functional responses of waxy alleles were different based on their specific polymorphic sites. These research groups discovered a G/T SNP (Chen et al., 2008; Dobo et al., 2010) at the splicing donor site of intron 1 (Isshiki et al., 1998) and three SNP mutations, namely, an A/G SNP (Hoai et al., 2014) in exon 4 (Larkin and Park, 1999; Mikami et al., 1999), an A/C SNP (Chen et al., 2008; Dobo et al., 2010) in exon 6 (Wang et al., 1995; Cai et al., 1998; Larkin and Park, 2003; Mikami et al., 2008), and a C/T SNP (Chen et al., 2008; Dobo et al., 2010) in exon 10 (Wang et al., 1995; Hirano et al., 1996; Cai et al., 1998). A 23 bp insertion in exon 2 is important in waxy rice, resulting in the loss of GBSS function, which in turn causes the glutinous trait (Inukai et al., 2000; Wanchana et al., 2003; Teng et al., 2012). In parallel with waxy or glutinous rice, genetic identification and association mapping of the waxy gene in maize have been characterized in the recent decades (Huang et al., 2010; Hossain et al., 2019; Luo et al., 2020; Kim H. R. et al., 2021). The waxy gene of wild maize was located in chromosome 9 and comprised of 14 exons by 3.93 kb long (Luo et al., 2020), and has a higher genetic variation level than rice, representing most of the identified waxy mutations by insertions and deletions, such as *wx-m9, wx-m5, wx-B3, wx-m1*, and *wx-B4* (Huang et al., 2010). Up to the recent updates, despite the presence of several mutant waxy alleles (>50) in maize (Huang et al., 2010), a low number (≥ 10) of waxy alleles (*Wx*^*a*^, *Wx*^*b*^, *Wx*^*in*^, *Wx*^*mp*^, *Wx*^*mq*^, *Wx*^*op*/*hp*^, *Wx*^*Iv*^, *Wx*^*mw*^, *Wx*^*Ia*^, and *wx*) have collectively been discovered in rice genome (Cai et al., 1998; Larkin and Park, 2003; Wanchana et al., 2003; Liu et al., 2009; Yang et al., 2013; Zhang et al., 2021; Zhou et al., 2021), and summarized for the identification of their specific polymorphic sites carrying different functional properties (Zhang et al., 2021). Not only being limited to these functional waxy alleles (*Wx*) these days, many researchers have been increasing their interests on other starch synthesis-related genes (SSRGs), like *SSII*/*ALK* (alkali degeneration) genes for the improvement of ECQ rice (Huang et al., 2020, 2021).

In our findings, we observed 12 non-synonymous substitutions (fSNPs) in coding exons 2, 4, 6, 9, 10, 12, and 13. Of these, three fSNPs, namely, A/G in exon 4, A/C in exon 6, and C/T in exon 10, were found in temperate japonica and indica, were in agreement with previous findings, and confirmed the strong association of SNPs with a waxy gene function. Cultivated haplotypes (ScR-2 and ScR-4) also showed an A/C SNP in exon 6 in tropical japonica, aromatic, and admixed accessions (Figure 2), whereas 10 C/T SNPs (exon 10) were found in indica and tropical japonica accessions, represented by the ScR-1 haplotype. These findings assume that the aromatic accessions were closely related to the japonica accessions during A/C substitution and that the admixed and indica accessions were closely related to tropical japonica accessions by C/T polymorphism. An analysis of the SNPs and InDel variations within the *GBSSI* gene region for haplotyping of 3,000 rice accessions revealed 134 fSNPs (non-synonymous mutations) and 27 InDels, representing 139 haplotypes along chromosome 6 (Supplementary Data 4). After filtering major haplotypes, there were three major haplotypes, each haplotype covering more than 500 rice accessions, namely, Hap_2, Hap_3, and Hap_4, with respective rice accession numbers of 765, 791, and 562. Of these SNPs, four fSNPs, G/A and A/G in exon 4, A/C in exon 6 (one of the major haplotypes found in the 3K rice accessions), and G/A in exon 12, coincided with our findings (Supplementary Figure 5).

A 23 bp duplication (insertion) was identified as a unique coding sequence in exon 2 of the waxy gene, originating from tropical glutinous rice, and it can explain the lack of amylose and *Wx* protein, resulting in chain termination (Wanchana et al., 2003). Our results also revealed a 23 bp insertion (exon 2) in Te_J-2 and ScR-4, as well as the Ref. haplotype, representing japonica, indica, and wild rice (Figure 1). Additionally, we found five fSNPs, namely, four G/As (exons 2, 9, and 23) and one A/C (exon 13), representing their belonged haplotypes. Interestingly, all these five fSNPs were observed only in wild rice. These alleles may be mutations limited to the wild group because domestication can be independent during the process of rice evolution (Konishi et al., 2006). Although the wild group and cultivated group were similar in terms of independent domestication of the *GBSSI* gene, functional waxy alleles could be identified for each haplotype. There was also a key point in separating glutinous rice from non-glutinous rice as the *Wx*^*a*^ allele can enhance the level of *GBSSI* compared with that for the *Wx*^*b*^ allele, resulting in a high AC in the grain (Cai et al., 1998). Furthermore, *Wx*^*a*^ is mainly present in indica rice, whereas *Wx*^*b*^ is primarily found in japonica rice (Tian et al., 2009). Our findings of functional haplotypes showed that non-synonymous substitutions mostly belonged to *Wx*^*a*^ allele, particularly in the wild haplotypes, where all showed *Wx*^*a*^ allele by G nucleotide at G-T splicing site of intron 1.

Resistant starch (RS) content in rice is controlled by *GBSSI*-encoded waxy gene (Kong et al., 2015), which is also responsible for different rice ACs (Ayres et al., 1997; Bao et al., 2006; Chen et al., 2008; Teng et al., 2013), and their positive correlation (between RS and AC) was confirmed by high *R*^2^-value (>75%) (Bao et al., 2017). We measured AC of cultivated rice accessions, classifying different groups of ecotypes, and we found the japonica groups in lower ACs (17.35 ± 4.42 and 18.00 ± 4.37) and higher AC was observed in indica (19.77 ± 3.59) and aus (20.64 ± 2.96) (Supplementary Table 4, Supplementary Figure 6). This finding was consistent with the previous findings of one research group (Bao et al., 2017) that the RS content of aus was significantly higher than those of remaining ecotypes, except indica. In a comparison of two major ecotypes (japonica and indica), the AC of indica was significantly higher than that of japonica (Supplementary Figure 6B), which was also the same trend of AC variation reported by Bao et al. (2017). According to the classified haplotypes, the identified fSNPs of cultivated haplotypes were separately located from those of wild haplotypes (Figure 1). However, many research groups have diagnosed that a SNP (G/T) located at the splicing site of intron 1 is a waxy allele, which is responsible for amylose biosynthesis (Ayres et al., 1997; Bao et al., 2006; Chen et al., 2008; Teng et al., 2013). The exact position of this allele was chr06_1765761 of *GBSSI* gene, and it can be considered as *Wx*^*a*^ in indica and *Wx*^*b*^ in japonica (Bao et al., 2017). Our haplotyping revealed 15 haplotypes (8 cultivated and 7 wild) indicating G/T SNPs in the same position (chr06_1765761). All wild haplotypes expressed *Wx*^*a*^ waxy allele, whereas three of cultivated haplotypes represented *Wx*^*b*^ by their responsible nucleotide G or T at intron 1 splice site. These findings of functional haplotypes in line with waxy alleles can be future determiners for the identification of AC levels, which have in turn to be considered in the development of new rice varieties.

Plant or animal domestication is one of the most important events in human history because of the need to consider food security to support growing populations. Domestication is often associated with a reduction in genetic variation in domesticated plants compared to their wild types (Konishi et al., 2006). Nucleotide diversity is a measure of genetic variation associated with other statistical measures of population diversity (Kilian et al., 2007). Our results suggested a domestication signature of *GBSSI* in cultivated rice, especially in japonica ecotypes or bred varieties by their considerable low diversity values (Figure 3, Supplementary Figure 3). In terms of nucleotide diversity, the upstream of this gene was under selective sweep due to a lower diversity feature than that of downstream region. The tail region of *GBSSI* was under selective sweep, which seemed to be made by recent selection because all the classified rice groups had the same feature. Low nucleotide diversity came through not only selective sweep but also a bottleneck effect. These findings were assumed to support the previous findings of a lower genetic diversity level by a genetic bottleneck effect, which were experienced in the domestication selection of waxy locus detected in waxy rice genome (Olsen and Purugganan, 2002; Yamanaka et al., 2004; Wei et al., 2012). On the other hand, indica and admixture were higher in nucleotide diversity after the wild. In general, a lower diversity index indicates a smaller nucleotide difference among the samples. None of the cultivated subgroups had high nucleotide diversity compared with the wild group. This is also possible because of a strong artificial selection during the domestication of the *GBSSI* gene, which in turn indicates the high genetic diversity level in the wild ancestors together with the absence of segregating sites in the cultivated rice (Yamasaki et al., 2005).

Tajima's *D* test has been employed for years to estimate genetic diversity by measuring the allele frequency to infer natural selection under the influence of population history (Tajima, 1989). Using the SNP data, our calculated Tajima's *D* values for the wild group and different classified groups of cultivated rice (Figure 4, Supplementary Figure 4) revealed that only the wild varieties after bred showed a negative Tajima's *D* value compared with the landrace and weedy varieties, which indirectly represented ecotypes such as aus, indica, and admixed. This was assumed to be because of the excess of low-frequency haplotypes composed of rare alleles compared to expectations under a neutral model of evolution (Zhou et al., 2016). In both analyses, Tajima's *D* generally showed the similar results. The wild rice was close to zero, indicating that it had almost the same observed and expected diversity, which seemed to be subjected under selection pressure. On the other hand, the bred variety showed the lower observed diversity than expected, which could be seen as an evidence of a strong purifying selection, or in another word, domestication. However, the fact that Tajima's *D* value of the region closer to the *GBSSI* gene was greater than that of other regions, indicating that it underwent a relatively accelerated selection, which may mean an active intervention in the *GBSSI* gene by humans in the process of domestication. On the other hand, weedy had a higher value than landrace, which means that weedy was subjected to a balancing selection after escaping from the bred variety, which might be subjected to breeding and then subjected to a deleterious selection by humans. An indication of balancing selection in ecotype-based analysis, indica and admixture ecotypes were signified by their high positive Tajima's *D* compared to others. On the other hand, both japonica ecotypes have undergone the population expansion by purifying selection, indicating a normal bottleneck event by considering the population survival. The wild can also be under the same selection with indica and admixture ecotypes by similar diversity and Tajima's *D* values. In brief, two different direction selections contributed to the domestication of the waxy gene i.e., *GBSSI*.

A simple and an effective process for assessing the genetic variability of a large number of germplasms is clustering (Belamkar et al., 2011) by statistical approaches, such as population structure analysis (Pritchard et al., 2000) and PCA (Rohlf, 2000). Despite the mixed structures of internal subgroups of varieties or ecotypes, we could observe a clear separation of varieties or ecotypes at most of the *K*-values. Similar admixed patterns were also found to be not only among the classified cultivated subgroups but also by admixing with the wild. However, greater genetic isolations indicated by *F*_*ST*_ values were clearly noticed between any cultivated variety or ecotype and wild. Moreover, in case of ecotypes, both PCA and *F*_*ST*_ values between temperate japonica and aus as well as aromatic and aus were interestingly isolated. Such inconsistent findings were assumed because of a much larger differentiation in the population size of temperate japonica than that of aus and aromatic rice ecotypes. Two major ecotypes in our study, indica and temperate japonica, had a moderate genetic differentiation by *F*_*ST*_ value 0.470, compared to their individual distances from the wild, 0.271 by indica and 0.703 by temperate japonica.

A wide range of genetic distances represented by the fixation index (*F*_*ST*_ values) have been reported in previous studies, such as 0.87 for an elite rice genotype from Chile (Becerra et al., 2015), 0.86 for Ugandan rice (Mogga et al., 2018), and 0.01–0.76 for African rice (Ndjiondjop et al., 2018). In this study, the *F*_*ST*_ values of wild rice with landrace, weedy, and bred rice were relatively larger compared to those among the three variety types (Figure 2C). However, in the cultivated groups, the values were low, indicating a close association to support the result of population stratification of landrace, weedy, and bred rice with separate patterns. For ecotypes, the greatest genetic distance was found between temperate japonica and aus ecotypes (0.747), and the lowest was found for the admixed-indica pair (−0.136) (Table 2). According to the standard of Wright, the values more than 0.25 indicate a high genetic differentiation (Wright, 1984), so most (>65%) of our findings in this study indicate a relatively high genetic differentiation.

About 59 haplotypes created a network, representing different genetic variants of *GBSSI*. We observed close associations of the cultivated haplotypes with each other, and their relatedness with wild haplotypes was low. Two major cultivated ecotypes, temperate japonica and indica, shared their genetic similarities only in major haplotype, Hap_1 and their pure haplotypes were associated from one another within the same ecotype. Most of the wild haplotypes were dispersed throughout the network. Some wild accessions also shared similar genetic variants with indica and japonica by Hap_1 while fewer wild haplotypes were connected in lower mutational steps by temperate japonica of Hap_1 and also with some indica haplotypes. These findings contradicted the fact that the highest number of wild rice accessions harbored the first two major haplotypes of *GBSSI* in the haplotype network analysis (Singh et al., 2017). Our findings showed the higher nucleotide diversity of wild together with functional haplotypes identified for new fSNPs, which can be future functional waxy alleles for *GBSSI*.

To develop new cultivars in good ECQ through breeding program, knowledge in genetic diversity, genetic relationships, and population structure is important. Simultaneously, the genetic basis of agronomic traits is also a scientific issue in determining crop promoting breeding programs (Pasam et al., 2012). In line with the two most commonly used methods in traits identification: (1) QTL mapping for crossed results (Skøt et al., 2005) and (2) association mapping between molecular markers and traits (Verdeprado et al., 2018), the breeding program is vital to identify the genes of interest for their efficient performance on agronomic traits (Kim M.-S. et al., 2021). A marker-assisted backcross selection employed by MABc was the most recent breeding method that not only potentially overcome conventional breeding limitations (Suh et al., 2013) but it can also speed up the genome recovery (99%) of recurrent-parent within three backcrosses (Miah et al., 2015). A recent successful MABc program for ECQ rice was introduced through the functioning of *SSIIIa* gene responsible for low-AC, and the breeding program was mainly performed based on the identified fSNP (G/A) located on exon 3 of *SSIIIa* gene (Kim M.-S. et al., 2021). For the waxy gene, some functional alleles have long been measured for their performances in ECQ-related breeding development: G/T (at splice site of intron 1) introgression in Chinese male sterile hybrid rice (LTF-A and ZS-A) (Liu et al., 2006) and CT_17_ transfer into II-32B lines for its low-AC (Jin et al., 2010). Chen et al. (2008) also concluded that the three gene haplotypes represented for a combination of two waxy SNPs (G/T in intron 1 and A/C in exon 6) were suitable for the selection of desired apparent AC (AAC) rice breeding program. Our findings on new functional waxy SNPs from the wild haplotypes were seemed to be useful markers for future breeding program of new glutinous rice varieties improvement.

## Conclusions

This study has given an account of collective and supportive information about functional variants, genetic diversity, and genetic distance (relationship) within the *GBSSI* gene region for 475 accessions of the Korean rice collection. The haplotype analysis revealed the information on the genetic variants of *GBSSI* alleles together with their corresponding haplotypes. The three previously reported functional waxy alleles, namely, G/A in exon 4, A/C in exon 6, and C/T in exon 10, could be identified through their domestication only in cultivated subpopulations, and simultaneously, additional new wild waxy alleles (four G/A, A/G, and T/C) were detected, indicating that the wild and cultivated rice groups independently arose based on the *GBSSI* waxy allele function. Moreover, it seemed to be that the cultivated rice accessions were not hybridized with any wild rice for the purposes of glutinous rice production or that no wild rice was grown in Korea. Despite these findings, the wild accessions with the higher nucleotide diversity can serve as potential donors of functional waxy alleles under a directional balancing selection.

Evolutionary analyses revealed the genetic diversity of *GBSSI* in 475 rice accessions and its genetic distance and relatedness between and among the populations. In this study, domestication signatures were obviously indicated by a diversity reduction using the classified cultivated subgroups compared to the higher nucleotide diversity of wild rice. Different Tajima's *D* values of cultivated rice groups were additional indicators for *GBSSI* evolution under different selective sweeps, indicating the *GBSSI* gene region under selective sweep. In spite of complicated associations between and among the cultivated subgroups and wild in PCA, clearly differentiated cultivated varieties or ecotypes were stratified as separate populations in PCA. Greater isolations of cultivated subgroups from one another and genetic distance of each subgroup from the wild were clearly indicated by their inferred *F*_*ST*_ values. Through these our findings, further research program on breeding of waxy rice can be promoted.

## Data Availability Statement

The datasets presented in this study can be found in online repositories. The names of the repository/repositories and accession number(s) can be found at: NCBI with accession number (BankIt2477403: MZ514142–MZ514616).

## Author Contributions

TZM and Y-JP: conceptualization. S-HC and I-MC: methodology. TZM, J-MY, and S-HC: investigation. TZM and K-WK: data curation and writing—review and editing. TZM and J-MY: writing—original draft preparation. Y-JP: project administration. All authors have read and agreed to the published version of the manuscript.

## Conflict of Interest

The authors declare that the research was conducted in the absence of any commercial or financial relationships that could be construed as a potential conflict of interest.

## Publisher's Note

All claims expressed in this article are solely those of the authors and do not necessarily represent those of their affiliated organizations, or those of the publisher, the editors and the reviewers. Any product that may be evaluated in this article, or claim that may be made by its manufacturer, is not guaranteed or endorsed by the publisher.
