# Supplementary figures and images for: Haplotype Variations and Evolutionary Analysis of the Granule-Bound Starch Synthase I Gene in the Korean World Rice Collection
Source: Front Plant Sci. 2021 Aug 24;12:707237. doi: 10.3389/fpls.2021.707237 (PMC8421862; doi:10.3389/fpls.2021.707237)

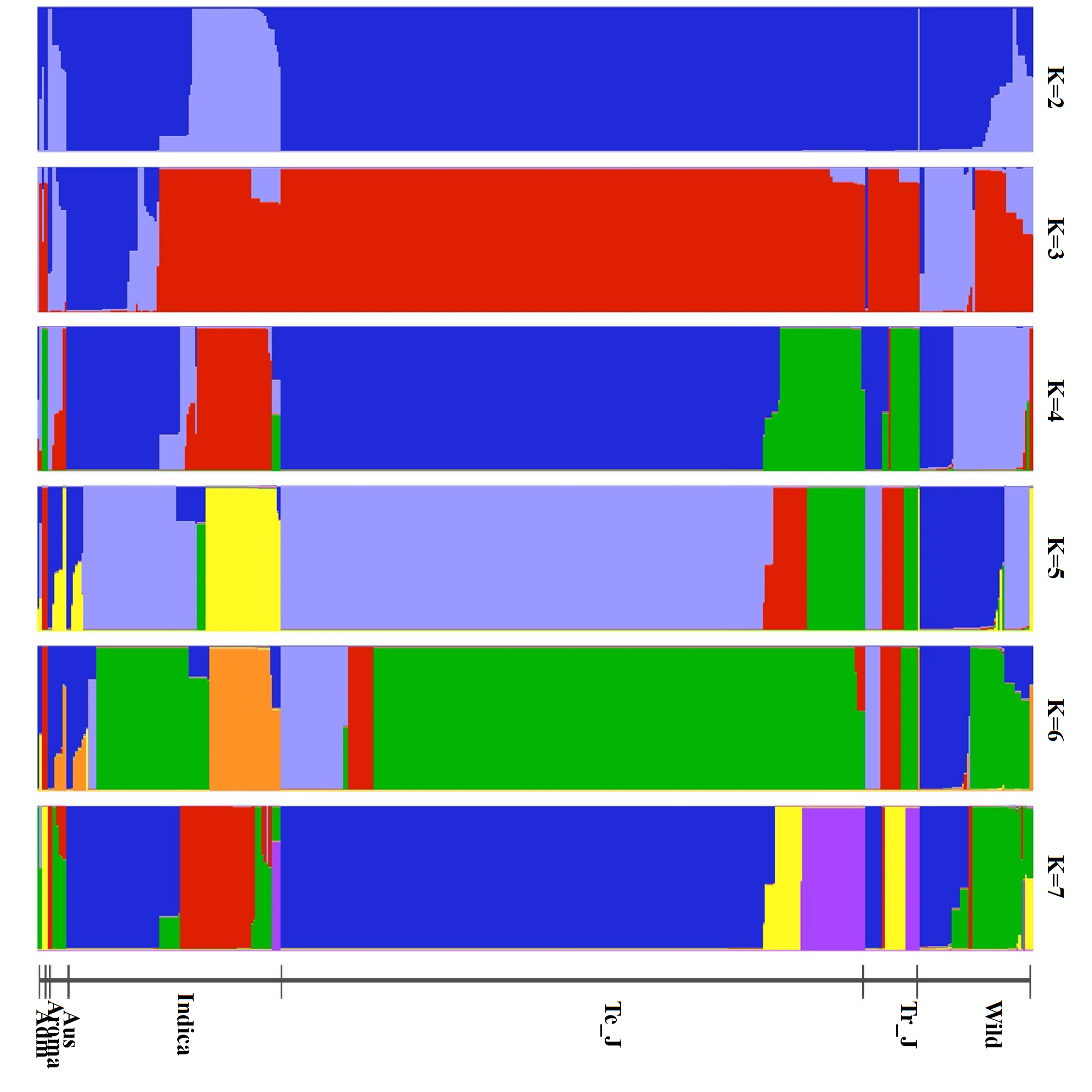

Supplement: Supplementary file 1 [file Image_1.TIF]

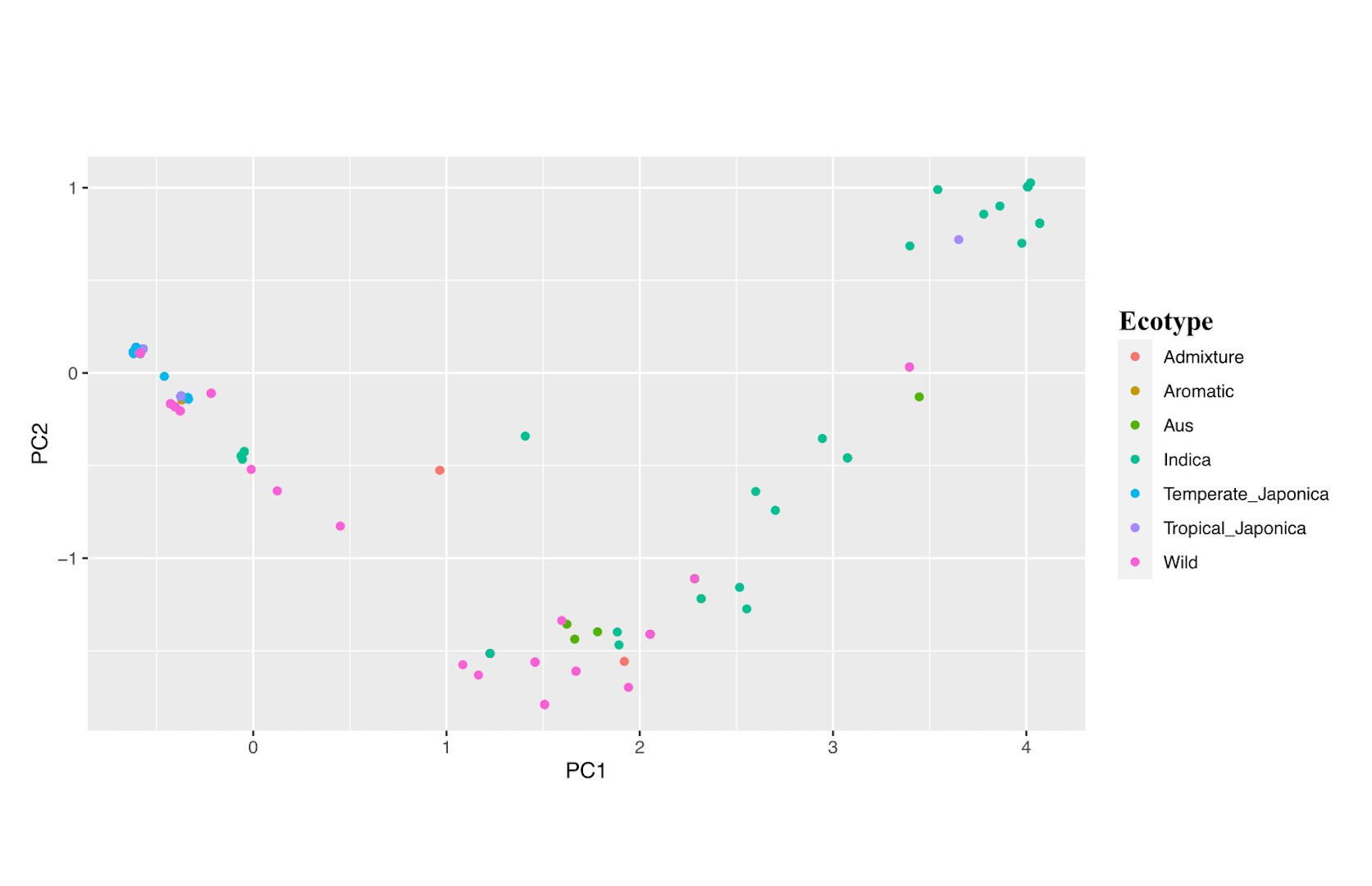

Supplement: Supplementary file 2 [file Image_2.TIFF]

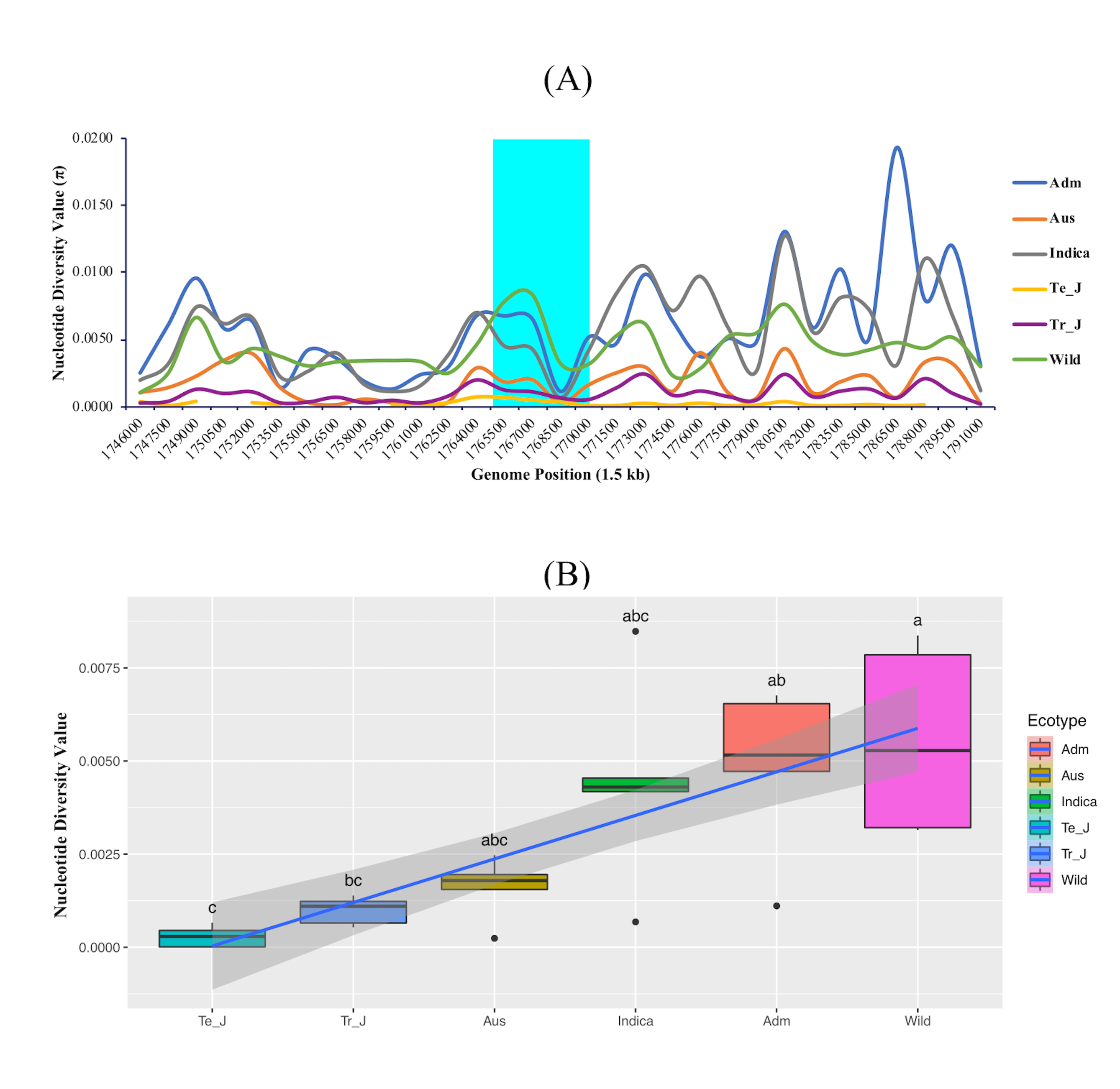

Supplement: Supplementary file 3 [file Image_3.TIFF]

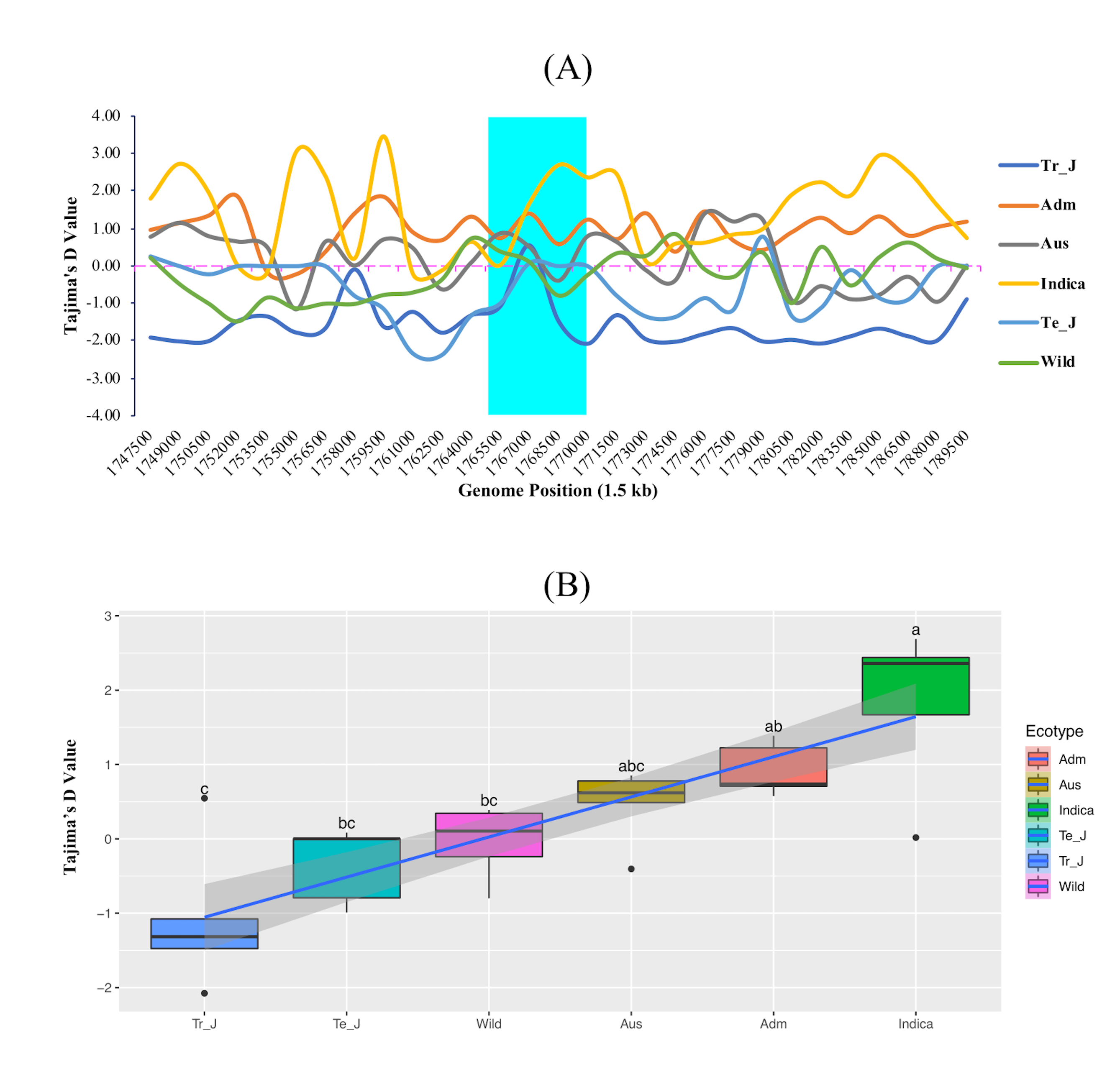

Supplement: Supplementary file 4 [file Image_4.TIFF]

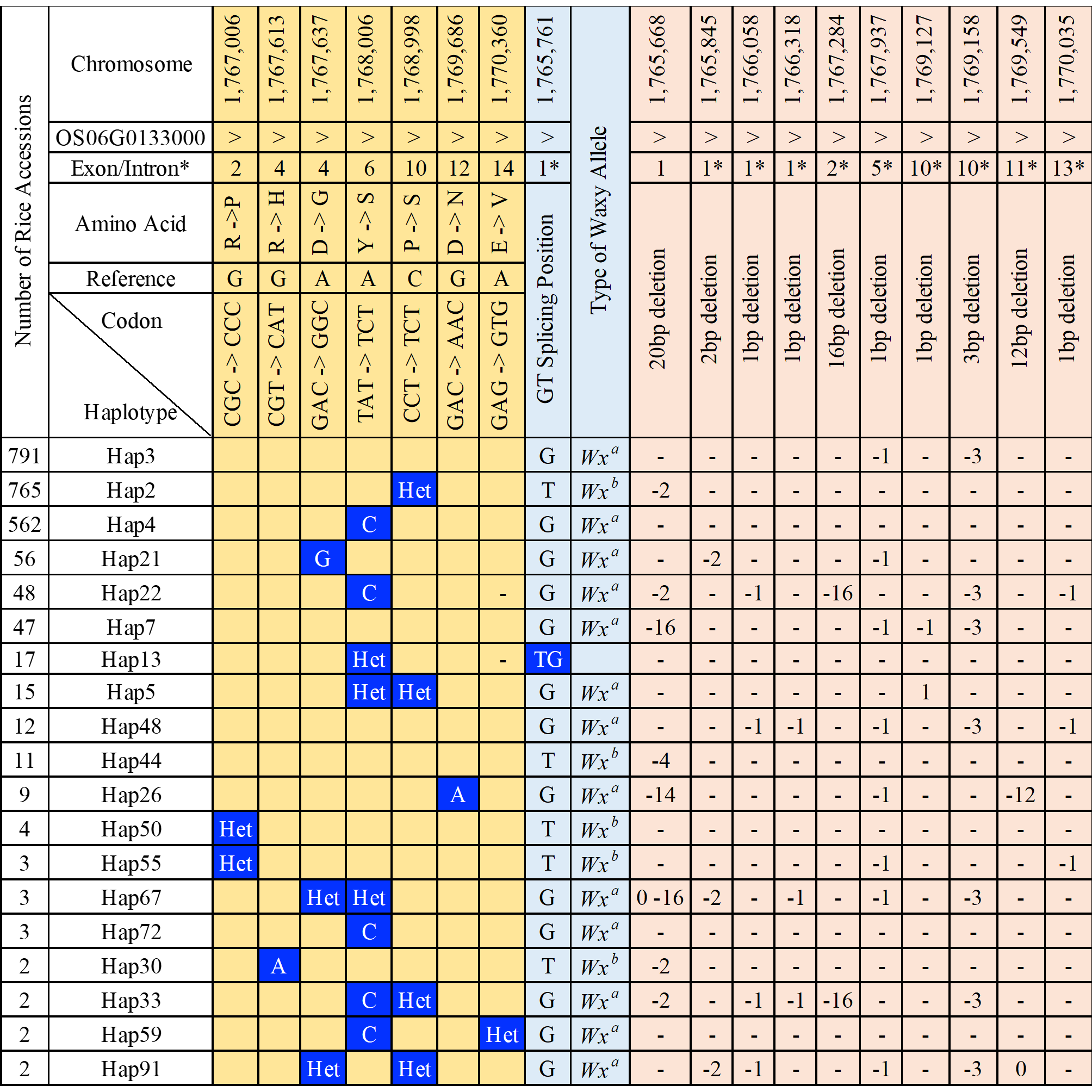

Supplement: Supplementary file 5 [file Image_5.TIF]

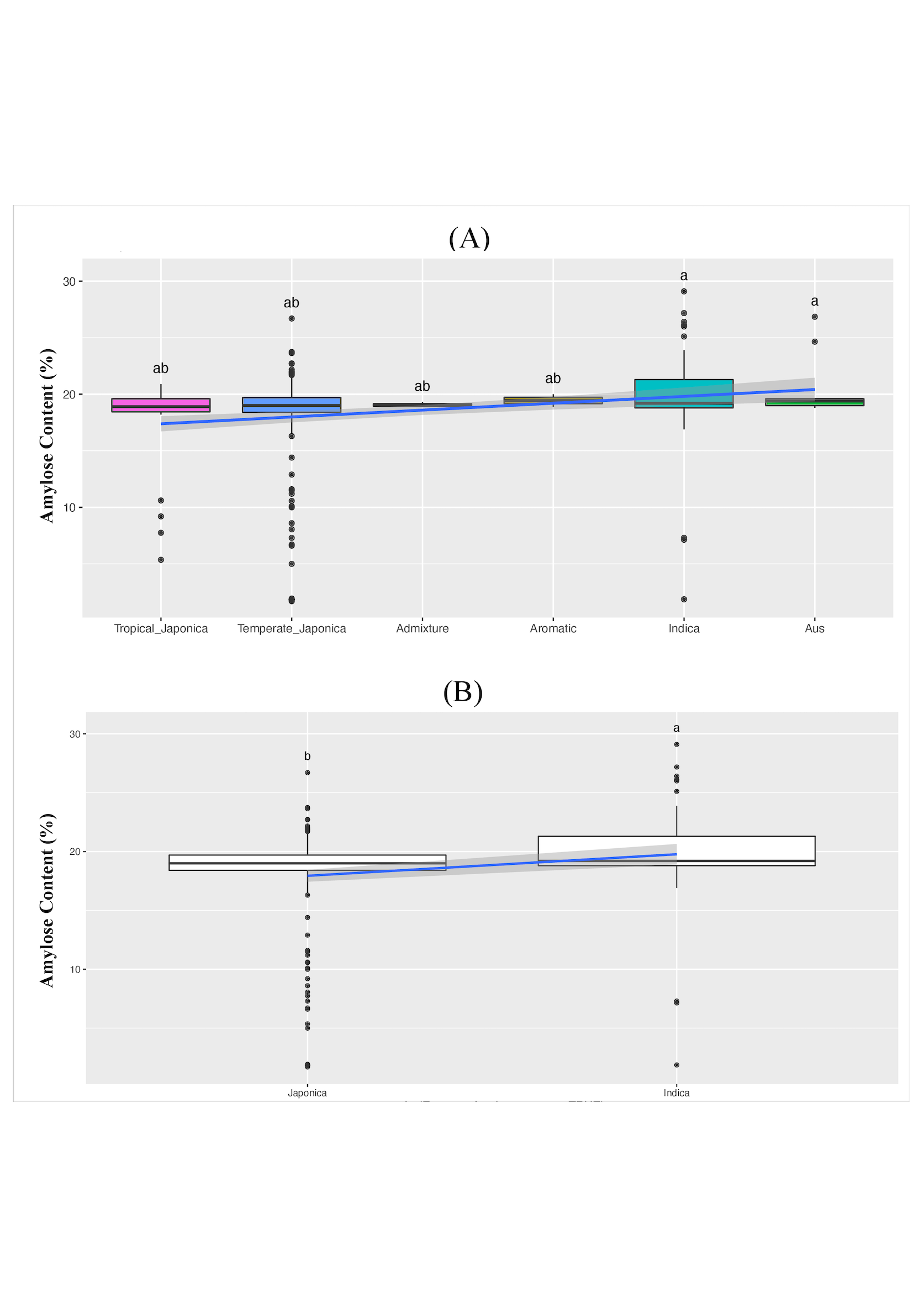

Supplement: Supplementary file 6 [file Image_6.TIFF]
