# Supplementary material for: Haplotype Variations and Evolutionary Analysis of the Granule-Bound Starch Synthase I Gene in the Korean World Rice Collection
Source: Front Plant Sci. 2021 Aug 24;12:707237. doi: 10.3389/fpls.2021.707237 (PMC8421862; doi:10.3389/fpls.2021.707237)
Supplement: Supplementary file 11 [file Data_Sheet_1.docx]

***Supplementary Materials***

***Supplementary Data***

**Supplementary Data S1**. Passport information of 475 KRICE_CORE (Korean World Rice Collection) rice accessions used in this study.

**Supplementary Data S2**. List of 475 accessions of Korean rice collection indicating raw data for different genetic variants (SNPs and InDels) within the gene region of *GBSSI* by haplotyping. Originally, we found a total of 59 haplotypes, representing 371 positions by SNPs (single nucleotide polymorphism) and InDels (Insertions or Deletions) detected in both exons and introns including UTR (untranslated regions) of 5' and 3'. Columns for codon and amino acid (AA) were supported for the positions where SNP substitutions in exons were identified.

**Supplementary Data S3**. Haplotyping revealed a list of 59 haplotypes including all 43 SNPs (single nucleotide polymorphism) (12 Non-synonymous and 31 Synonymous) and 70 InDels (insertions or deletions) detected in the gene region of *GBSSI* in 475 accessions of Korean rice collection. In this data, detected variants (SNPs and InDels) were summarized only from exons. Condon changes together with amino acid transition due to SNP substitutions were also supported at each position of coding regions.

**Supplementary Data S4**. List of 139 haplotypes generated from haplotyping analysis of 3000 rice within the gene region of *GBSSI.*

***Supplementary Tables***

S**upplementary Table 1**. Summary of 475 accessions of Korean world rice collection based on their accession numbers by means of different types

| Varietal Type | No. of Rice Accessions Occupied | | Ecotype |
| --- | --- | --- | --- |
| Landrace | 49 | 45  20  9  2  1 | Temperate Japonica  Indica  Tropical Japonica  Aus  Admixture |
| Weedy | 28 |  |  |
| Bred | 320 | 227  70  14  5  2  2 | Temperate Japonica  Indica  Tropical Japonica  Aus  Aromatic  Admixture |
| Wild | 54 |  |  |
| Unknown | 24 | 7  12  3  2 | Temperate Japonica  Indica  Tropical Japonica  Aus |
| Total | 475 | 475 |  |

**Supplementary Table 2.** Summary of average nucleotide diversity (π) and Tajima’s D values within *GBSSI* gene region of 475 accessions of Korean world rice collection by means of different variety types

| Varietal Type | Parameter | Value |
| --- | --- | --- |
| Landrace | Nucleotide Diversity (π value) | 0.0036 |
|  | Tajima’s D value | 0.2764 |
| Weedy | Nucleotide Diversity (π value) | 0.0033 |
|  | Tajima’s D value | 1.5286 |
| Wild | Nucleotide Diversity (π value) | 0.0056 |
|  | Tajima’s D value | -0.0403 |
| Bred | Nucleotide Diversity (π value) | 0.0013 |
|  | Tajima’s D value | -1.0488 |

**Supplementary Table 3.** Summary of average nucleotide diversity (π) and Tajima’s D values within *GBSSI* gene region of 475 accessions of Korean world rice collection by means of different ecotypes.

| Ecotypes | Parameter | Value |
| --- | --- | --- |
| Temperate Japonica | Nucleotide Diversity (π value) | 0.0003 |
|  | Tajima’s D value | -0.3401 |
| Tropical Japonica | Nucleotide Diversity (π value) | 0.0010 |
|  | Tajima’s D value | -1.0801 |
| Indica | Nucleotide Diversity (π value) | 0.0044 |
|  | Tajima’s D value | 1.8349 |
| Aus | Nucleotide Diversity (π value) | 0.0016 |
|  | Tajima’s D value | 0.4665 |
| Admixture | Nucleotide Diversity (π value) | 0.0049 |
|  | Tajima’s D value | 0.9264 |
| Wild | Nucleotide Diversity (π value) | 0.0056 |
|  | Tajima’s D value | -0.0403 |

**Supplementary Table 4.** Descriptive statistics for the mean value of amylose content among the classified subpopulations of 360 Korean world rice collection (KRICE_CORE)

| **Ecotype** | **Mean ± SD** | **Median** | **Data Range** | **IQR** |
| --- | --- | --- | --- | --- |
| Temperate Japonica | 18.00 ± 4.37 | 19.00 | 1.70 – 26.70 | 1.30 |
| Tropical Japonica | 17.35 ± 4.42 | 18.90 | 5.36 – 20.90 | 1.15 |
| Indica | 19.77 ± 3.59 | 19.20 | 1.87 – 29.10 | 1.50 |
| Aus | 20.64 ± 2.96 | 19.40 | 18.80 – 26.85 | 0.60 |
| Aromatic | 19.45 ± 0.78 | 19.45 | 18.90 – 20.00 | 0.55 |
| Admixture | 19.07 ± 0.21 | 19.00 | 18.90 – 19.30 | 0.20 |

*SD: Standard Deviation;* *IQR: Interquartile Range*

***Supplementary Figures***

**Supplementary Figure 1.** Population structure clustering of the *GBSSI* gene in 475 accessions of the Korean rice collection analyzed by increasing K values from 2 to 7. For each K value, different colors refer to different numbers of clustered populations.

**Supplementary Figure 2.** Two dimensional (2D) principal component analysis (PCA) of 475 accessions of the Korean rice collection.

**Supplementary Figure 3.** Nucleotide diversity analysis of *GBSSI* (*Os06g0133000*) in 475 accessions of Korean rice collection by means of ecotypes (temperate japonica, tropical japonica, indica, aus, aromatic and admixture) and wild. **(A)** Nucleotide diversity (π-value), representing the number of nucleotide variations at individual segregating sites in 1.5 kb sliding windows within the *GBSSI* gene region. Cyan indicates the *GBSSI* gene region, and each colored line represents a different rice subpopulation. **(B)** Box plots representing the different distribution patterns of *GBSSI* genetic variations based on mean nucleotide diversity values among the subpopulations.

**Supplementary Figure 4.** Tajima’s D values of *GBSSI* (*Os06g0133000*) in 475 accessions of the Korean rice collection by means of ecotypes (temperate japonica, tropical japonica, indica, aus, aromatic and admixture) and wild. **(A)** Tajima’s D values, representing different individual segregating sites in 1.5 sliding windows of the *GBSSI* gene region. Cyan indicates the *GBSSI* gene region, and each colored line represents a different rice subpopulation. **(B)** Box plots represent different distribution patterns of *GBSSI* genetic variations according to Tajima’s D values among the subpopulations.

**Supplementary Figure 5**. Analysis on haplotype variation of *GBSSI* gene in 3000 (3K) rice accessions. In fact, there were a total of 139 haplotypes, but, in this figure, only the major haplotypes and some haplotypes having more than 2 accessions were summarized to check how many haplotypes together with their variations (SNP or InDel) were shown the same to those of our findings. Chromosome positions were based on the observed variations (SNPs or InDels) within the selected haplotypes. Light-blue colored columns indicate corresponded waxy allele based on the possession of “G” or “T” nucleotide at GT splicing site of Intron 1. Blank cell indicates major alleles in that position. “Het” refers to “heterozygote”, and a dash (-) indicates the position for an “unknown” nucleotide or generally refers to “N” (any nucleotide A, T, G or C).

**Supplementary Figure 6.** Statistical analysis on the measured values of amylose content in 360 Korean rice collection by Scheffé test. The resulted mean values were compared based on the classified rice groups (ecotypes) determined by 5% significant level (**P* < 0.05). The test was performed **(A)** among all the classified ecotypes and **(B)** within two major ecotypes (indica and japonica).
